# The Development and Concurrent Validity of a Multi-Sensor-Based Frailty Toolkit for In-Home Frailty Assessment

**DOI:** 10.3390/s22093532

**Published:** 2022-05-06

**Authors:** Chao Bian, Bing Ye, Alex Mihailidis

**Affiliations:** 1 Institute of Biomedical Engineering, University of Toronto, Toronto, ON M5S 1A1, Canada; alex.mihailidis@utoronto.ca; 2 Toronto Rehabilitation Institute, University Health Network, Toronto, ON M5G 2A2, Canada; bing.ye@utoronto.ca; 3 Department of Occupational Science and Occupational Therapy, University of Toronto, Toronto, ON M5S 1A1, Canada

**Keywords:** frailty, sensors, measurement, validity, internet of things, smart home, telehealth

## Abstract

Early identification of frailty is crucial to prevent or reverse its progression but faces challenges due to frailty’s insidious onset. Monitoring behavioral changes in real life may offer opportunities for the early identification of frailty before clinical visits. This study presented a sensor-based system that used heterogeneous sensors and cloud technologies to monitor behavioral and physical signs of frailty from home settings. We aimed to validate the concurrent validity of the sensor measurements. The sensor system consisted of multiple types of ambient sensors, a smart speaker, and a smart weight scale. The selection of these sensors was based on behavioral and physical signs associated with frailty. Older adults’ perspectives were also included in the system design. The sensor system prototype was tested in a simulated home lab environment with nine young, healthy participants. Cohen’s Kappa and Bland–Altman Plot were used to evaluate the agreements between the sensor and ground truth measurements. Excellent concurrent validity was achieved for all sensors except for the smart weight scale. The bivariate correlation between the smart and traditional weight scales showed a strong, positive correlation between the two measurements (r = 0.942, *n* = 24, *p* < 0.001). Overall, this work showed that the Frailty Toolkit (FT) is reliable for monitoring physical and behavioral signs of frailty in home settings.

## 1. Introduction

According to *World Population Prospects: the 2019 Revision*, one in six people in the world will be over age 65 by 2050 (16%), up from one in 11 in 2019 (9%) [1]. Older adults are at a high risk of disability, long-term hospitalization, unfavorable discharge, and death after injury. However, age itself is a poor risk indicator because of the heterogeneity of older adults [2,3,4]. The concept of “frailty” identifies older adults with low physiological reserves, vulnerability to illness, and a high risk of disability, institutionalization, and death [5,6]. People in a state of frailty are vulnerable to stressful situations and gradual decline towards dependence if the frailty state is not detected early enough. Frailty will put an enormous burden on older adults and their family caregivers including impaired quality of life, loneliness [7], and increased healthcare costs [8]. The incremental 1-year healthcare costs associated with frailty went from CAD 10,845 to CAD 12,360 in Canada [9]. In Germany, progression from non-frailty to frailty caused healthcare costs to increase by 54% if three or four frailty symptoms were present, and 101% if five frailty symptoms were present [10].

Despite the adverse outcomes, there is an opportunity to prevent or remedy frailty [11]. Studies have shown that frailty is a dynamic process characterized by frequent transitions between frailty states over time [11,12]. Fried et al. defined three frailty states based on the number of phenotypic criteria that a person meets, such as slowness, weakness, or shrinking [6]. A person is frail if meeting three to five phenotypic criteria, pre-frail if meeting one or two criteria, and non-frail if none of the phenotypic criteria are present. The dynamic transitions between frailty states mean that pre-frail people can transition to non-frail with intervention. The likelihood of transition between frailty states depends highly on one’s preceding frailty state [11]. For older adults living with frailty, the probability of transitioning to the prefrail state decreases over time, while the probability of dying increases. Therefore, early detection is critical for preventing or reversing frailty.

Monitoring older adults’ behavioral changes could provide insight into how frailty develops [13]. Assessing these changes in real life may help identify frailty early [13]. Technologies may offer cost-effective opportunities for assessing frailty in older adults’ real lives, such as home environments. Recent developments in sensor technologies for health monitoring have shown opportunities to monitor frailty signs at home. Wearable sensors from different manufacturers (e.g., BioSensics, Newton, MA, USA; Shimmer, Dublin, Ireland; Actibelt, Munich, Germany) were used to measure frailty criteria such as muscle strength [14], gait [15], and physical activities [16]. Researchers also used wearable sensors during standardized physical tests, such as Timed Up and Go to assess frailty [17].

However, research on technologies for assessing frailty at home is still in its infancy. Most existing systems focused on a single dimension of the multidimensional frailty and only on homogenous sensors such as wearables [18,19,20]. Very few studies used non-contact ambient sensors that can be set up in living environments to assess frailty [21]. Many frailty criteria and early signs associated with frailty were not studied using heterogeneous technologies in home settings. Examples of these criteria or early signs include sedentary behavior [22], constricted life space [13], declined Activities of Daily Living (ADLs) (e.g., stair climbing) [23], or self-report exhaustion [6]. Many of these criteria are measurable by off-the-shelf sensors. For example, wearable sensors, such as Fitbit [23] or force sensors [24], can assess sedentary behavior. Accelerometers and GPS [25] can measure life-space mobility. Moreover, inertial sensors [26,27,28] or cameras [29] can measure ADLs such as chair stand or stair climbing. Additionally, previous studies have used speech-recognition technology to capture health data for cancer, diabetes, or heart failure [30]. Similar technology could also collect self-report data for assessing frailty.

Moreover, while Internet connectivity and remote access should become an essential design requirement for remote health monitoring systems [31,32], most existing systems for assessing frailty lack telecommunication capability, making the systems not convenient or scalable for real-life home deployment. Modern technologies such as the Internet of Things (IoT) and Cloud Computing have demonstrated their capabilities to overcome the remote access barrier in many health care applications (e.g., telemedicine, medical imaging, public health, and patient self-management) [33]. 

This paper introduces a home-based frailty assessment system named Frailty Toolkit (FT). FT adopts the following design principles to fill some of the gaps identified above:

1. FT consists of a unique and new collection of heterogeneous customized sensors that use off-the-shelf sensing modules. FT was tailored for home-based frailty assessment by measuring early behavioral signs of frailty under free-living conditions in older adults’ daily lives. The system does not require older adults to perform standardized physical tests, such as gait speed tests or chair stand tests, which could need extra supervision and compliance to perform correctly. FT consists of a unique and new collection of heterogeneous off-the-shelf sensors tailored for home-based frailty assessment. The sensors measure early behavioral signs of frailty in older adults’ daily lives.
2. FT uses state-of-the-art cloud services from major commercial cloud service providers. The cloud technologies enable real-time telecommunication and the use of advanced data analytics. The use of cloud services can benefit research data analysis for this study and future real-world deployment of similar systems in terms of implementation process and lessons learned.
3. FT makes no use of cameras or wearable sensors. Such devices are perceived to have privacy and obtrusiveness concerns, undermining user acceptance. Instead, ambient sensors require minimal effort from end-users to monitor frailty. Users are not required to carry any device.
4. The design of FT involves older adults’ perspectives from the beginning to enhance its usability.

The overall objective of FT research is to design and develop a multi-sensor-based system for use in homes and evaluate the system’s effectiveness in assessing frailty. The specific objective of this paper is to validate the concurrent validity of measuring frailty criteria using multiple sensors in a simulated home lab environment. Analyzing habits or daily patterns related to frailty was not the focus in the current stage. 

The rest of this article is structured as follows: Section 2 presents the design method and experiment protocol. Section 3 reports the statistical results. Section 4 discusses our study with guidance for future work. Finally, Section 5 presents the conclusions.

## 2. Materials and Methods

### 2.1. Frailty Criteria and Sensor Selection

The sensor selection for FT was based on frailty criteria defined in existing clinical frailty scales and signs that were significantly associated with frailty by previous studies. We used Fried’s frailty phenotype model [6] as the primary reference clinical model. We also referenced the cycle of frailty theory [34] and phenotypes that are significantly associated with frailty, such as life-space mobility [13] and ADLs [23]. The goal of the design was not to include all related frailty criteria but certain highly related ones that can be measured by sensors meeting the design principles above. Certain frailty criteria were not included due to not satisfying the design principles described above. For instance, despite being an effective criterion for identifying frailty [35], walking speed measurement requires a wearable, a vision-based sensor, or a mat sensor (e.g., GAITRite) [36,37]. When used in unsupervised home settings, these sensors are subject to difficult user acceptance, declining adherence, and privacy concerns [38,39,40,41]. The design of the FT focused on ambient sensors without using cameras or wearable sensors. FT also does not require a fixed space from home settings for using the GAITRite-like mat sensor. The selection of frailty criteria aims to build a new frailty assessment paradigm by combining selected frailty criteria from different models tailored for ambient-based frailty assessment under free-living conditions. Accordingly, we can identify effective off-the-shelf sensing modules to measure those criteria or phenotypes and customize the sensors for FT using the sensing modules. We chose ambient sensors instead of wearable or vision-based sensors because they are non-invasive and can preserve privacy.

#### 2.1.1. Strength

**Figure 1:**
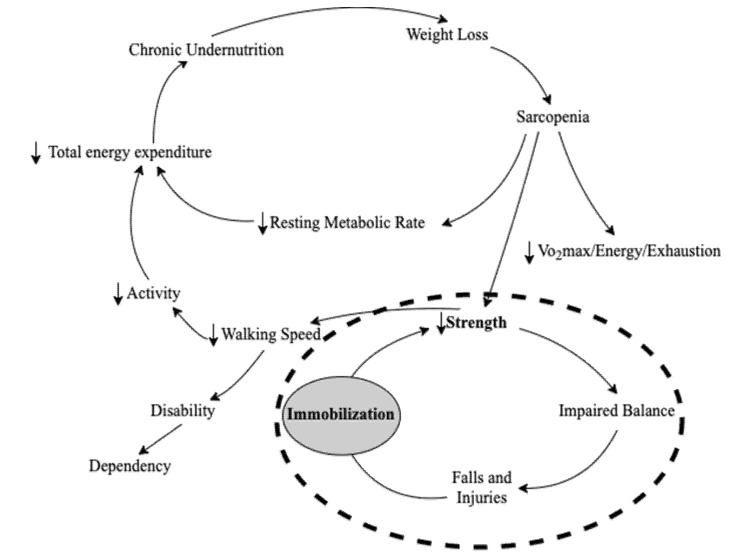
The cycle of frailty, adapted with permission from Ref. [29]. Copyright 2022 Elsevier.

Handgrip strength was one of the five phenotypes defined in Fried’s frailty model. However, the hand dynamometer’s absence of remote communication, inadequate user-friendliness, and rare availability even in primary care settings limit its usage in the home by older adults without assistance [42]. To find a home-friendly sensor to measure strength, we looked into the cycle of frailty theory shown in Figure 1, which defines a progressing cycle and a broader range of interrelated frailty phenotypes that include the five criteria in Fried’s phenotype model. We found that strength and immobilization belong to the same progressing cycle (see Figure 1). Immobilization is the exogenous cause of sarcopenia characterized by progressive and generalized loss of skeletal muscle mass and strength [43,44,45,46]. Moreover, another study found that immobilization was associated with sedentary behavior [47]. As sedentary behavior is associated with frailty [22], it may be an alternative underlying strength indicator for off-the-shelf sensors to measure at home. We, therefore, designed a mat sensor to monitor sedentary behavior instead of straight measuring handgrip strength. Equation (1) calculates the sedentary duration.

Sedentary duration, s = t_unoccupied_ − t_pre_occupied_ (1)

where:

t_unoccupied_ = the time when a mat sensor detects an unoccupied seat
t_pre_occupied_ = the time when a mat sensor detects a previously occupied seat

In addition to sedentary behavior, strength can also be measured by physical performance tests such as standing balance, chair stand, and stair climbing [48]. Among these tests, stair climbing time was significantly associated with the early onset of frailty [23]. A frail person would take a longer time to climb stairs compared with a non-frail person [23]. Similar to sedentary behavior, stair climbing can also be measured by off-the-shelf sensors more easily than handgrip strength in an unsupervised home environment if a flight of stairs is available. In this study, we chose to use ultrasonic distance sensors to detect human presence on a flight of stairs for calculating stair climbing time. We used two distance sensors and placed them at the first and last step of a flight of stairs to capture the start and end of a stair-climbing event. Equation (2) calculates the stair climbing time.

Stair climbing time, sc = t_distance_sensor2_ − t_distance_sensor1_ (2)

where:

t_distance_sensor1_ = the time when the first distance sensor detects a person’s proximity
t_distance_sensor2_ = the time when the second distance sensor detects a person’s proximity

#### 2.1.2. Self-Report Exhaustion

A customized Raspberry Pi-based smart speaker collects the self-report exhaustion data. The smart speaker was programmed to collect users’ speech through a 6-mic audio board (ReSpeaker 6-Mic Circular Array Kit for Raspberry Pi, Seeed Technology Inc., Shenzhen, China). The smart speaker used a customized cloud-based Amazon Lex chatbot to manage the conversation with its users. Amazon Lex is an Amazon Web Service that provides automatic speech recognition and natural language understanding technologies to create a Speech–Language Understanding system or a chatbot. The Lex chatbot was built to ask two questions from the Center for Epidemiological Studies-Depression (CES-D) scale [49] for the self-report exhaustion data collection. 

**Figure 2:**
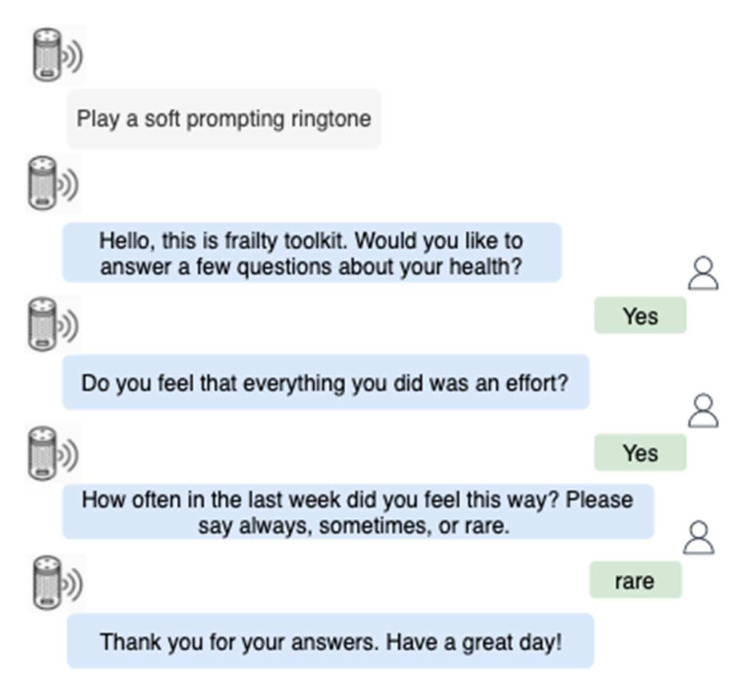
Sample smart speaker conversation.

Unlike the commercial smart speakers, such as Google Home or Amazon Echo, that must be triggered using a keyword from a user, the smart speaker in this study was developed to be easier to use by older adults. Firstly, the smart speaker was programmed to proactively initiate conversations and ask questions without using a wake-up word from older adults. The conversation would be initiated when other sensors in FT detected a person. Secondly, the smart speaker only asked three questions that accepted single-word answers. The first question was an availability confirmation question that asked users if they were available to answer questions at the moment. If the user answered “yes”, the following two questions were asked sequentially: “Do you feel that everything you did was an effort, or you could not get going in the last week?” and “How often in the last week did you feel this way?”. Answer options (e.g., yes or no) were prompted to the users following each question. The prompts guided older adults to choose the right words to minimize potential misunderstandings in the conversation when answering the questions. While the first two questions were a “yes” or “no” question, the third question expected users to answer one of three words: “always”, “sometimes”, or “rare”. Users who responded “sometimes” or “always” were categorized as frail for this criterion. Lastly, the AWS Lex chatbot was programmed with a fallback plan to repeat questions if the first attempt was not successful. A sample interaction between the smart speaker and a user is illustrated in Figure 2. 

#### 2.1.3. Physical Activity

Fried’s phenotype model uses calorie consumption per week to measure physical activity. Technologies such as smartwatches or computer vision can measure calorie consumption or estimate consumption by identifying activities or food [50,51]. However, these technologies either require high compliance from users or violate user privacy. Instead of measuring calorie consumption, we proposed to use motion sensors to measure gross movement at room levels through the number of sensor triggers. Information such as presence duration in a room (e.g., bedroom, living room) and frequency of room transitions can be obtained. An earlier study has shown the potential of using room transition data to distinguish frailty statuses [52]. The study used Bluetooth beacons placed in each room and a smartphone carried by users to detect room transitions. This study used motion sensors as they may be a more effortless and unobtrusive alternative to capture similar information. Each functional room has one motion sensor installed. The room presence duration can be calculated using the Equation (3). In addition, the mat sensor can also be used to provide additional information on low physical activity, as immobilization described in Section 2.1.1. (Strength) is related to low activity in Figure 1.

Room presence duration, r = t_next_motion_sensor_ − t_pre_motion_sensor_, (3)

where:

t_next_motion_sensor_ = the first confirmed time when the next motion sensor detects a person
t_pre_motion_sensor_ = the first confirmed time when the previous motion sensor detects a person.

#### 2.1.4. Weight

Weight loss can be easily measured by tracking weight changes using a bathroom scale. To facilitate the use of a low-cost home bathroom scale, we modified a standard digital bathroom scale by adding an Arduino-based microcontroller with a wireless communication module and a LED light. With the modification, the scale can initiate and prompt the weight measuring process by working with other FT sensors and sending weight data to the IoT platform. For example, once the motion sensor in the bathroom detects a person’s presence, the smart weight scale would receive a command from the motion sensor immediately to blink the LED light prompting the start of the weight measuring process. The smart weight scale was factory calibrated as the weighing module was not modified.

#### 2.1.5. Life Space Mobility

Life space is one of the behavioral precursors of frailty, as a large cohort study found that women who left the neighborhood less frequently were 1.7 times more likely to become frail than those who left the neighborhood four or more times per week [13]. To estimate life space from home without using wearable technology such as GPS, we used a simple door sensor installed at the entrance door to monitor home entry and exit. Thus, parameters associated with life space, such as frequency and duration of being away from home within a specific time frame, can be estimated. The outing duration can be calculated using the Equation (4). However, as not every door event indicates a real outing, a confirmed outing can be determined by combining the door event with information from other sensors as shown in the Equation (5). Table 1 shows the final selection of sensors.

Outing duration, od = t_door_event_ − t_pre_door_event_ (4)

where:

t_door_event_ = the time when a door sensor detects a door opening event
t_pre_door_event_ = the time of a previous door opening event
  (5) $$\mathrm{Outing},\;o=\left\{\begin{array}{cc} 1, & \;\;\mathrm{if}\;\mathrm{od}>5\;\min\;\mathrm{and}\;\mathrm{no}\;\mathrm{events}\;\mathrm{from}\;\mathrm{other}\;\mathrm{sensors}\\ 0, & \;\;\mathrm{if}\;\mathrm{od}\leq 5\;\min\;\mathrm{or}\;\mathrm{events}\;\mathrm{from}\;\mathrm{other}\;\mathrm{sensors} \end{array}\right.$$

### 2.2. User-Centred Design

Deploying technologies into older adults’ homes faces many challenges caused by older adults’ physiological impairments, stigma concerns, obtrusiveness, or technical barriers [53]. The technology design process should involve older adults and other stakeholders to understand their living habits and home environments, their preferred ways of interacting with the technologies, and their preferred functionalities and deployment process [53]. We conducted focus group interviews with older adults before the start of the design [54] and adopted the following practical design recommendations learned from the focus groups. 

Each sensor in FT operated autonomously after powering on. No further interventions were needed. The software running on the sensors could be updated remotely without user interaction. The only requirement from the users was to recharge the batteries or plug in the power adapter, which was found acceptable by older adults [54]. Long battery life was essential for the long-term use of health monitoring devices, with a minimum of 1 week considered ideal [55]. Low-power sensors and microcontrollers enable sensors in the toolkit to run for months before recharging. For instance, a similar passive infrared motion sensor can last at least 3 months using two AA batteries [56]. This minimal effort from the users was positioned to reduce the user-perceived complexity of the system and potential operating errors.

Moreover, the smart speaker in FT could be placed in a convenient location according to the older adults’ lifestyles. For example, some older adults preferred to interact with the speaker in the kitchen while preparing food. Others chose to put the speaker near the bed for a quick conversation before sleeping. In addition, to enhance usability, the smart speaker would play a soft prompting ringtone before the frailty conversation, resembling the one used in the airport before any announcements. At the end of the conversation, the speaker would confirm all information was received and appreciate users’ responses. Additionally, we gave users’ feedback on the smart weight scale by using an LED light to show weight measurement progress. When the IoT platform received the weight data successfully, the LED light turned green to tell the user that the measurement was complete. An audio prompt can be added in the future iteration of the development to further enhance usability for older adults with sight problems.

Other design considerations that have not been incorporated into the current system but will be in the following design iteration include: (1) Adding physical buttons to the smart speaker for those older adults who prefer the familiar, simple button press to the verbal conversation with the smart speaker. The goal here was to reduce the complexity or probability of confusion to enhance usability. (2) Using existing technologies such as smartphones to improve data collection and communication. The use of smartphones, in this case, does not mean older adults have to carry smartphones as the earlier studies did [57,58]. Instead, the under-used smartphone or other existing technologies at home could be reused to enhance data collection in a user-familiar way, such as text messaging or app notification. For instance, an AI-powered text messaging chatbot can ask self-report questions and obtain user responses by text messages. Information about frailty can also be shared by text messages or an app notification. (3) Adding warming functionality into the mat sensor to enhance technology enjoyment. (4) Adding enjoyable functions to the smart speaker, such as playing music and telling jokes [54]. 

### 2.3. Protocol

As this study aims to validate the sensor measurements but not frailty assessment results, a convenient sample of healthy young participants is sufficient to achieve the goal. A convenience sample of nine healthy, young adults was recruited by sending group emails to research labs and posting flyers in hospitals of the University Health Network (UHN), Toronto. The recruitment lasted from May to August 2021. Participant’s inclusion and exclusion criteria are as follows:

Inclusion criteria

1. Minimum 18 years old;
2. Able to understand and speak English;
3. Able to give informed consent;
4. Able to attend at least one experiment session.

Exclusion criteria

1. Have trouble getting in and out of bed without assistance;
2. Have trouble walking or always use a wheelchair;
3. Cannot speak due to speech impairment;
4. Cannot hear due to hearing impairment.

**Figure 3:**
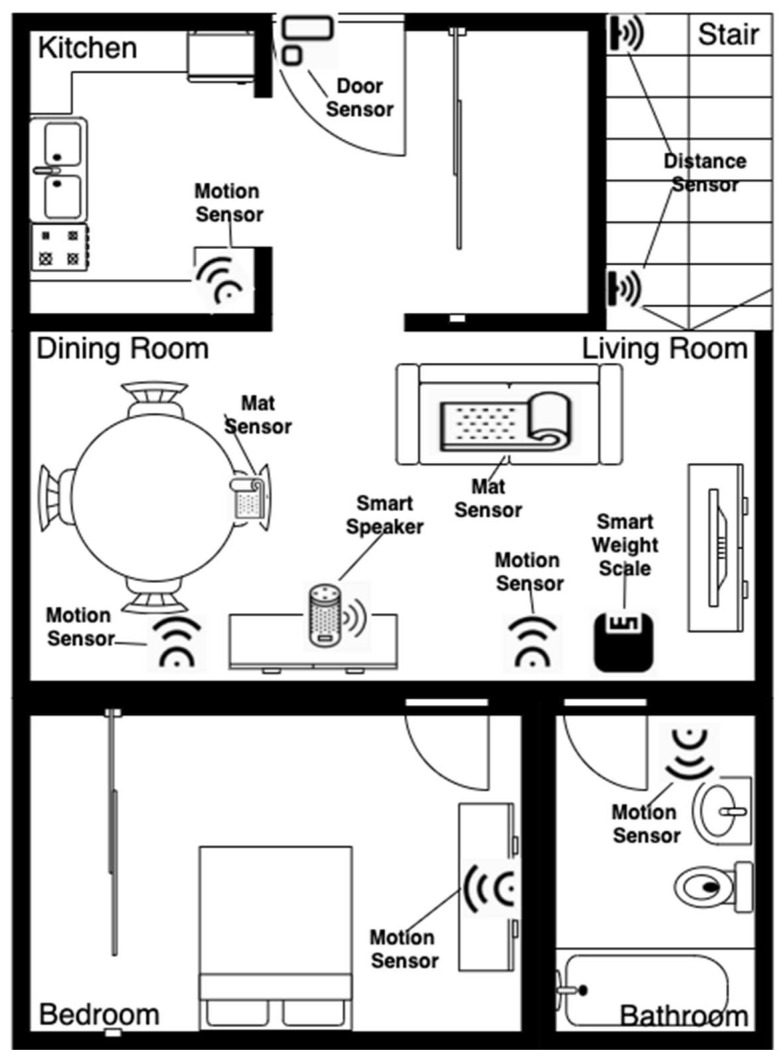
Sensor setup layout in HomeLab.

Participants’ demographic information was collected to determine their eligibility. Each participant attended one experiment session in a simulated home called HomeLab at KITE Research Institute of Toronto Rehabilitation Institute, UHN, Toronto, Canada. The HomeLab is a “home within a lab”, resembling a typically furnished single-story one-bedroom apartment with functional plumbing and wiring. Figure 3 illustrates the HomeLab layout and sensor setup.

Each participant performed a series of daily activities during the experiment session to test pre-installed sensors in HomeLab. These activities included room transitions (to test motion sensors), main entrance entry and exit (to test the door sensor), stair climbing (to test distance sensors), weight measuring (to test the smart weight scale), sitting (to test the mat sensor), and exhaustion question answering (to test the smart speaker). 

Each participant completed three runs of the experiment. Each run of the experiment took at least 15 min. In the first run, each participant was given detailed verbal instructions by a researcher (CB) on the activities to be performed. The instructions included going to a particular room (e.g., living room), measuring weight using the smart weight scale, climbing a flight of stairs, sitting on a chair with a mat sensor, and conversing with the smart speaker. Participants were then asked to perform similar activities based on their own decision and pace in the second and third runs. A summary of the experimental protocol is shown in Table 2. Participants were required to perform the activities defined in the protocol at least once. It was expected that participants would repeat these activities multiple times. These activities triggered the sensors to generate sensor data. Depending on the sensor type, the data could contain information about the room where a participant is present (e.g., living room or dining room), weight, occupancy status for the first or last step of the flight of stairs, chair occupancy, or door opening status. All data were transmitted to a cloud-based IoT platform and immediately timestamped when stored in a cloud database attached to the IoT platform. The sensor data were then transferred to a server at the UHN, Toronto, Canada. 

**Figure 4:**
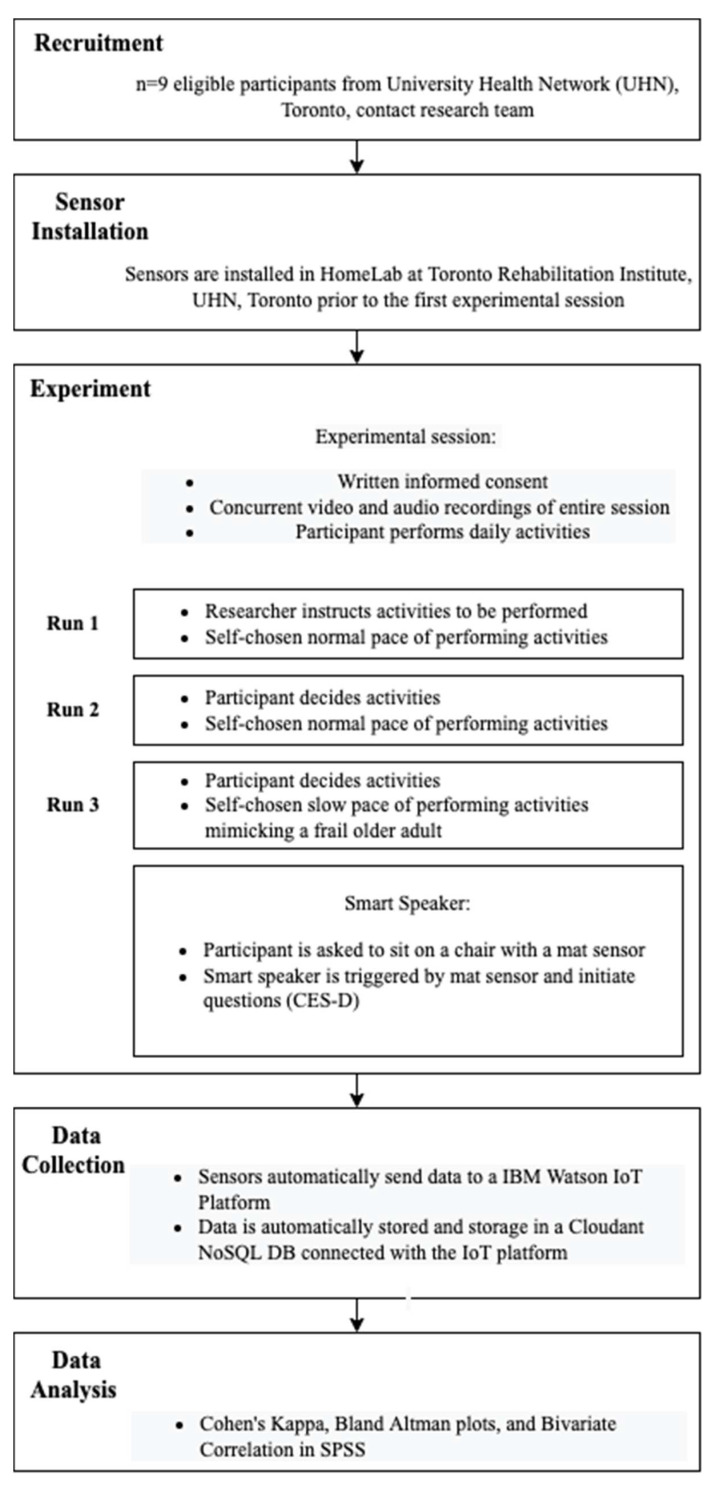
The schematized protocol for the experimentation.

All experiment sessions were video and audio recorded. The video and audio recordings were used to extract ground truth data for the activities performed by each participant except for the weight. For instance, the ground truth data for the room-level physical activity were the room presence in each room in HomeLab for an individual. The weight ground truth data were manually collected using a traditional non-digital weight scale in HomeLab at the end of each session. All activities were performed under the supervision of a researcher (C.B.). During the experiment, the researcher was not in HomeLab but stood at a catwalk overhanging around the lab with a bird’s-eye view of the lab. Break periods were preserved between different test runs. A schematized protocol for the experimentation is shown in Figure 4. 

### 2.4. Data Processing

The sensor measurement for each frailty criterion was compared with the corresponding ground truth measurement (i.e., video or audio recordings, traditional mechanical weight scale). The agreements between the two approaches were assessed using the Cohen’s Kappa test and Bland Altman plots. The Cohen’s Kappa test was used for categorical data from the motion sensor. The Bland Altman plots were used for continuous data, such as sedentary time and stair climbing time. Bivariate correlation was used to analyze weight data. All statistical tests were performed by the Statistical Package for Social Sciences (SPSS Inc., Chicago, IL, USA). The interpretation of agreement strength for Cohen’s Kappa was following Landis and Koch’s guidelines (1977) [59]. Cohen’s Kappa was calculated using the Equation (6).

Cohen’s Kappa, k = (p_o_ − p_e_)/(1 − p_e_) (6)

where:

p_o_ = the relative observed agreement between the sensor and ground truth measurement
p_e_ = the hypothetical probability of chance agreement

For Bland Altman plots, more than 20% of the value that fell outside the limits of agreement (LoA) would be considered no agreement between the two methods. *p* values less than 0.05 were considered to be statistically significant. The upper and lower LoA were calculated using the Equations (7) and (8).

Lower Limit = Mean of difference − (1.96 × Standard deviation of difference)(7)

Upper Limit = Mean of difference + (1.96 × Standard deviation of difference) (8)

Once the sensor measurements are validated, the toolkit will be tested with older adults living with and without frailty to validate clinical effectiveness in detecting frailty. We plan to use the sensor data to train classic machine learning models such as logistic regression, tree-based classifiers, and support vector machines. Models’ sensitivity and specificity will be estimated. 

## 3. Results

The three repeated runs of the experiment from nine participants generated 580 activities in total. 

### 3.1. The Smart Speaker for Self-Report Exhaustion

The smart speaker recognized and recorded participants’ answers to the two questions from the CES-D Depression scale. The recognized answers by the smart speaker had a 100% agreement with those recorded in the audio recordings (*n* = 27).

### 3.2. The Motion Sensor for Room-Level Physical Activity

Cohen’s Kappa was used to analyze the agreement in room-level physical activity measurements between motion sensors and video recordings. The proportion of observations in agreement p_o_ = 0.9558 and the proportion in agreement due to chance p_e_ = 0.2882. According to the Equation (6), the two methods generated a kappa value of k = (0.9558 − (0.2882))/(1 − (0.2882)) = 0.938 which showed an almost perfect agreement. The kappa value was significantly different from zero (k = 0.938, *p* < 0.001).

### 3.3. The Door Sensor for Life-Space Mobility

The door sensor had an 87.5% successful rate in detecting a door entry/exit event. However, the five undetected door entry/exit events were caused by a technical issue in the door sensor and solved immediately during the experiment. Therefore, the accuracy reaches 100% when excluding these outliers. See Table 3 of the percentage agreement. 

### 3.4. The Mat Sensors for Sedentary Behavior

The Bland–Altman plot was used to assess the agreement of the data from the mat sensor (*n* = 42) and distance sensor (*n* = 38) with their corresponding ground truth data extracted from the video recordings. 

**Figure 5:**
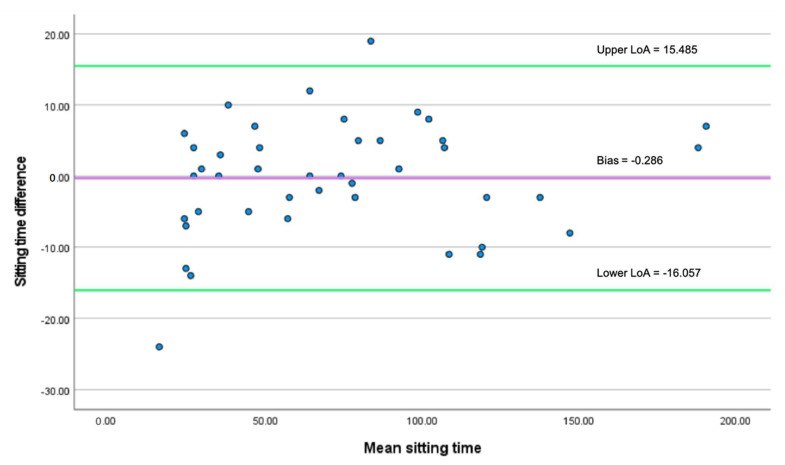
The Bland–Altman plot of agreement between mat sensor and video recording.

Figure 5 shows the plot of sedentary time duration between the two methods. The plot shows a mean difference, also called bias, of −0.286, presenting as a solid, purple line. The standard deviation is 8.046. According to the Equations (7) and (8), the upper LoA was calculated as −0.286 + (1.960 × 8.046) = 15.485. The lower LoA was calculated as −0.286 − (1.960 × 8.046) = −16.057. LoA is shown as the area between the two solid, green lines with 95% LoA. Only 4.8% (2 out of 42) values fall outside the LoA, suggesting a good agreement between the two methods [60,61]. As Figure 5 shows, the values are uniformly spread below and above the bias/mean difference within the LoA, suggesting no proportional bias. The result is also confirmed by linear regression (t = 1.097, *p* = 0.279).

### 3.5. The Distance Sensors for Stair Climbing Time

**Figure 6:**
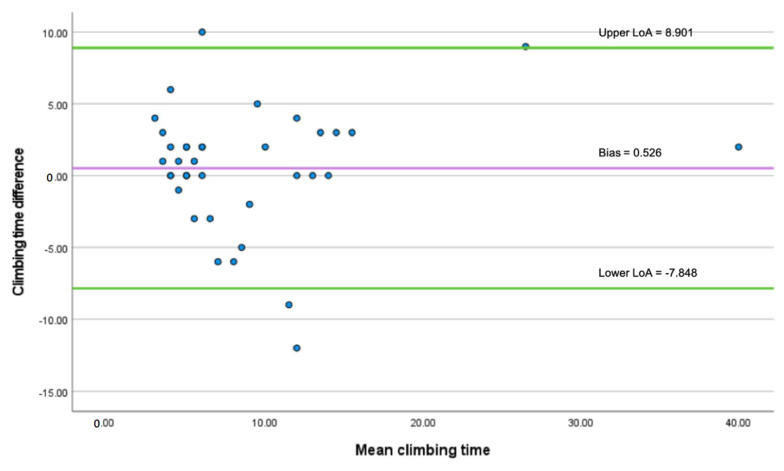
The Bland–Altman plot of agreement between distance sensor and video recording.

The distance sensor had a 50% success rate in detecting a stair-climbing event, including climbing up or down a flight of stairs, as shown in Table 4. The detected stair climbing time and ground truth time were compared using the Bland–Altman plot. Figure 6 reports a mean difference of 0.526 between the two measurements, shown as a solid, purple line. The standard deviation is 4.273. According to the Equations (7) and (8), the upper LoA was calculated as 0.526 + (1.960 × 4.273) = 8.901. The lower LoA was calculated as 0. 526 − (1.960 × 4.273) = −7.848. LoA is shown as the area between the two solid, green lines with 95% LoA. Most of the values are inside the LoA, suggesting a good agreement between the two methods. As Figure 6 shows, the values are uniformly spread below and above the bias within the LoA, suggesting no proportional bias. The result is also confirmed by linear regression (t = 0.653, *p* = 0.518).

### 3.6. The Smart Weight Scale for Weight

**Figure 7:**
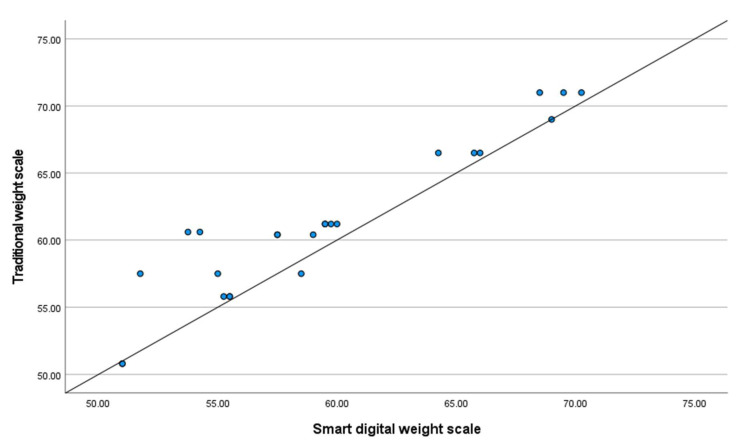
Scatterplot of the weight measured by traditional mechanical weight scale against smart digital weight scale.

There is a significant difference in the data generated from the two measurements. The difference could be caused by the incorrect operation or inaccurate readings of the traditional mechanical weight scale. However, bivariate correlation shows a strong, positive correlation between the two measurements, r = 0.942, *n* = 24, *p* < 0.001 (see Figure 7). This suggests that the smart weight scale can still track an individual’s weight change, but the measurement accuracy should be reexamined. 

## 4. Discussion

The FT has been designed to monitor frailty in home settings and assess it remotely before clinical visits. The toolkit helps identify frailty early, which can increase awareness of heightened vulnerability for older adults, allow for the institution of appropriate care plans, and allow for newly emerging evidence for the treatment of those who are frail to be readily implemented [62]. This work contributes a unique and novel selection of different ambient sensors and a customized smart speaker, extending the perspective of a home-based frailty assessment that was previously focused on IMU sensors and mobility-related parameters [63]. FT can therefore collect a broader set of behavioral and physical signs of frailty from older adults’ daily lives without being as intrusive as wearable or camera-based sensors. We intended to design the FT to require zero to minimal effort from older adults and passively monitor frailty from the living environment. The findings of this research will benefit clinicians, older adults, or family caregivers to remotely assess and monitor for frailty. FT can be used as a digital pre-clinical frailty screening tool at home by clinicians and older adults. It can also be used in collaboration with comprehensive clinical frailty assessment to monitor frailty progression and intervention effectiveness. 

This study is an essential foundational work in determining the system’s clinical effectiveness in assessing frailty. This phase of the research tested the concurrent validity of the sensor measurements of the proposed system. The statistical analysis showed that excellent measurement agreements were achieved for motion, mat, distance, and door sensors. This indicated that most of the sensors in the toolkit can generate correct readings for calculating frailty indicators, such as room presence duration, stair climbing time, and sedentary duration. However, there were missed detections from the motion sensors in the experiments. Most of the missed detections came from the first two participants. This problem turned out to be loose jumper wire connections that were not found in the dry run but revealed with the first two participants. After fixing the problem, no glitches were found for the rest participants. If the first two participants were excluded from the analysis, the agreements should be higher. 

Moreover, the distance sensor had only a 50% success rate in detecting a stair-climbing event. We found that the missed detection was because some participants used one side (e.g., left) of the stair to climb, whereas the sensors were installed on the opposite side (e.g., right). This caused the distance between the participants and the sensors to be beyond the distance threshold (60 cm), which did not trigger the sensors. In the future, different distance thresholds and mounting locations on the wall will be tested to find the ideal settings.

The smart weight scale did not show a good agreement with the traditional mechanical weight scale based on the Bland Altman plot method. As mentioned in the Results section, this lack of statistical agreement could be caused by inaccurate readings from the traditional weight scale during the experiments. However, the bivariate correlation between the two devices was strong and positive. The correlation coefficient of r = 0.942 is close to the one achieved (r = 0.993) in a previous study that tested the agreements between a modified bathroom scale and a traditional weight scale for frailty assessment purposes [64]. 

A highlight of the proposed system is the smart speaker. We successfully tested a customized smart speaker that uses context information that other ambient sensors provide to initiate conversations instead of a wake word required by commercial smart speakers. This is the first time a smart speaker was used for collecting self-report exhaustion data for frailty assessment. The role of the smart speaker in FT can be extended well beyond the data collection for self-report exhaustion. The smart speaker opens up new possibilities for collecting data from a multidimensional perspective around physical activity, food intake, life space, and more frailty indicators. The technical capabilities of the smart speaker also allow more functions to be added in the future. For example, more enjoyment features, such as playing music, can be integrated into the chatbot to enhance its usability. In addition, the smart speaker can be more helpful by giving users prompts or reminders about their health changes identified by FT and recommending interventions. 

One strength of the study is that the design of FT involved older adults’ voices from the beginning, which has not been found in similar technologies for assessing frailty. Flexible sensor placement, simplified human-computer interaction, and LED light prompting were some of the recommendations from the focus group study and were incorporated into the FT design. Another strength from the technical perspective is that we successfully implemented and tested the “trigger-action” mechanism between the IoT sensors to improve system usability and enhance the precision of data collection for particular sensors, such as the smart weight scale and smart speaker. With the mechanism, triggering a motion sensor or a mat sensor can further activate a smart weight scale, prompting the user for weight measuring or a smart speaker to initiate a conversation for collecting self-report exhaustion data, respectively. The sensor communication used the MQTT (MQ Telemetry Transport) protocol. This inter-sensor communication capability is the first time applied in similar systems. Lastly, participants were given opportunities to freely perform activities in HomeLab based on their paces. The freedom allowed the system to be tested in different scenarios closest to real life. 

FT also has some limitations. The testing was only done with young, healthy participants. It would have been more informative to have a comparison group of older participants tested in HomeLab. However, the recruitment was under COVID-19 restrictions to keep older adults safe. Moreover, as the objective of this study was to validate the sensor’s binary measurements but not the frailty assessment, the young, healthy participants should be sufficient. We took several measures to address the limitation from experiment protocol and system design perspectives. The study participants were asked to mimic common mobility characteristics of people living with frailty. These characteristics include slow walking speed, stair climbing, and longer sedentary behavior. Additionally, the smart speaker was designed to be easy to use for older adults. It initiated the conversation to bring the user’s attention. Only single-word answers (e.g., yes or no) for each of the three questions were required. The speaker was also programmed to prompt users with the expected answer options (i.e., “yes or no”, “always, sometimes, rare”). If the user missed the first attempt, the question would be repeated once again to provide another answer opportunity. Therefore, we believe it is very unlikely there would be a significant difference between young and older adults in responding to the questions asked by the smart speaker. Another limitation is that the FT system can only work for single-person dwellings as no sensors in the current FT can distinguish different persons. Indeed, the Radio Frequency Identification technology can be used to provide a unique ID for identifying different people. Computer vision technology can also provide personal identity through face recognition. However, both technologies can bring compliance or privacy concerns, making them not ideal for an application with older adults in home settings. Moreover, as FT is a proof-of-concept system, the current energy management for each sensor is not ideal for extending the battery life. The improvement in battery life can be made by removing unused electronic components on the current micro-controllers or using more energy-efficient micro-controllers. Furthermore, mat and distance sensors can only use time but not event signals to control their deep sleep mode. Unnecessary power waste using time-based sleep mode can shorten battery life. A comparator circuit with relays and logic gates could be one way to improve to allow pin interrupt-based sleep control.

Future work includes:

1. Test the system in HomeLab with non-frail and frail participants to validate the effectiveness of the frailty assessment. The test would also allow more data collection for training a machine learning model for classifying non-frail and frail older adults.
2. Move the system from the simulated home to an actual home of an older adult who lives alone. While older adults continue to live their own lives, the system will continuously run for a week to collect data. The data will be compared with the older adult’s frailty status measured by a reference clinical frailty scale.
3. Develop sensors to measure new frailty criteria or phenotypes in different domains and identify other persons living in the same household.
4. Compare sensors in FT with wearable sensors to validate the effectiveness of measuring certain frailty signs such as physical activity using ambient sensors.
5. Consult with clinicians to investigate how the data provided by FT would be made palatable and useful for them.

## 5. Conclusions

This study presented the design, development, and validation of a sensor-based toolkit for assessing frailty in home settings. The toolkit’s design focused on ambient sensing of behavioral and physical signs of frailty using different ambient sensors, a smart speaker, and a smart weight scale. The choice of heterogeneous sensors, a diverse set of frailty criteria, and the involvement of older adults’ perspectives in the design made this work unique from previous studies that mostly focused on wearable sensors and a single dimension of frailty. The prototype of the toolkit was deployed and tested in a simulated home lab. Statistical analysis of sensor data showed excellent concurrent validity for the ambient sensors and the smart speaker. The smart weight scale strongly correlated with its gold-standard measurement, but more validation is needed to confirm its concurrent validity. Overall, FT is reliable for monitoring physical and behavioral signs of frailty in home settings. The next step is to test the toolkit with older adults living with and without frailty to validate the system’s clinical effectiveness. Machine learning techniques will be used with the sensor data to build models and estimate classification performance for frailty.

## Figures and Tables

**Table 1:** Sensors’ hardware components, corresponding frailty criteria.

Sensor	Frailty Criteria
Mat sensor	Strength through sedentary behavior
Distance sensor	Strength through stair climbing performance (ADL)
Smart speaker	Self-report exhaustion
Motion sensor	Physical activity, life-space mobility (indoor)
Door sensor	Life space mobility (outdoor)
Smart weight scale	Weight

**Table 2:** Experiment Protocol for Testing the Sensors in FT.

Run	Run #1	Run #2	Run #3
**Run Type**	Guided, normal pace	Self-paced, normal	Self-paced, slow (mimicking frail older adults)
**Activity Type**	**Activities**		
Physical activity	Go to a room (e.g., living room) in HomeLab and do whatever activities in the room for 2 min.		
Sedentary behavior	Sit on a chair that has a mat sensor.		
Weight measuring	Measure weight using the smart weight scale.		
Stair climbing	Climb a flight of stairs.		
Self-report exhaustion	Have a conversation with the smart speaker.		
Life space	Enter or exit HomeLab through the main entrance door.		

**Table 3:** Frequency and percentage of successfully detecting door entry/exit event by the door sensor.

	Frequency	Percentage
**Detected**	35	87.5
**Undetected**	5	12.5
**Total**	40	100

**Table 4:** Frequency and percentage of successfully detecting stair-climbing events by the distance sensors.

	Frequency	Percentage
**Detected**	38	50
**Undetected**	38	50
**Total**	76	100

## Data Availability

The data presented in this study are available on request from the corresponding author. The data are not publicly available due to confidentiality protocol.
